# Antioxidant and Antimicrobial Activities of Chemically-Characterized Essential Oil from *Artemisia aragonensis* Lam. against Drug-Resistant Microbes

**DOI:** 10.3390/molecules27031136

**Published:** 2022-02-08

**Authors:** Khalid Chebbac, Hazem K. Ghneim, Abdelfattah El Moussaoui, Mohammed Bourhia, Azeddin El Barnossi, Zineb Benziane Ouaritini, Ahmad Mohammad Salamatullah, Abdulhakeem Alzahrani, Mourad A. M. Aboul-Soud, John P. Giesy, Raja Guemmouh

**Affiliations:** 1 Laboratory of Biotechnology Conservation and Valorisation of Natural Resources, Faculty of Sciences Dhar El Mahraz, Sidi Mohammed Ben Abdallah University, Fez 30000, Morocco; rajaguemmouh@yahoo.fr; 2 Department of Clinical Laboratory Sciences, College of Applied Medical Sciences, King Saud University, P.O. Box 10219, Riyadh 11433, Saudi Arabia; hghneim@ksu.edu.sa; 3 Laboratory of Biotechnology, Environment, Agri-Food and Health, Faculty of Sciences Dhar El Mahraz, Sidi Mohammed Ben Abdellah University, Fez 30000, Morocco; abdelfattah.elmoussaoui@usmba.ac.ma (A.E.M.); azeddin.elbarnossi@usmba.ac.ma (A.E.B.); 4 Laboratory of Chemistry, Biochemistry, Nutrition, and Environment, Faculty of Medicine and Pharmacy, University Hassan II, Casablanca 20000, Morocco; bourhiamohammed@gmail.com; 5 Laboratory of Natural Substances, Pharmacology, Environment, Modeling, Health and Quality of Life, Faculty of Sciences, Sidi Mohamed Ben Abdellah University, Fez 30000, Morocco; zineb.benziane@usmba.ac.ma; 6 Department of Food Science & Nutrition, College of Food and Agricultural Sciences, King Saud University, P.O. Box 2460, Riyadh 11451, Saudi Arabia; aabdulhakeem@ksu.edu.sa; 7 Toxicology Centre, University of Saskatchewan, Saskatoon, SK S7N 5B3, Canada; jgiesy@aol.com; 8 Department of Veterinary Biomedical Sciences, University of Saskatchewan, Saskatoon, SK S7N 5B3, Canada; 9 Department of Integrative Biology, Center for Integrative Toxicology, Michigan State University, East Lansing, MI 48824, USA; 10 Department of Environmental Science, Baylor University, Waco, TX 76706, USA

**Keywords:** essential oils, phytochemical analysis, antioxidant, antibacterial, antimicrobial resistance

## Abstract

This study investigated the chemical composition, antioxidant and antimicrobial activity of essential oil extracted from *Artemisia aragonensis* Lam. (EOA). Hydrodistillation was employed to extract EOA. Gas chromatography with flame ionization detection (GC-FID) and gas chromatography-mass spectrometry analyses (GC-MS) were used to determine the phytochemical composition of EOA. Antioxidant potential was examined in vitro by use of three tests: 2.2-diphenyl-1-picrilhidrazil (DPPH), ferric reducing activity power (FRAP) and total antioxidant capacity assay (TAC). Agar diffusion and microdilution bioassays were used to assess antimicrobial activity. GC/MS and GC-FID detected 34 constituents in the studied EOA. The major component was Camphor (24.97%) followed by Borneol (13.20%), 1,8 Cineol (10.88%), and Artemisia alcohol (10.20%). EOA exhibited significant antioxidant activity as measured by DPPH and FRAP assays, with IC_50_ and EC_50_ values of 0.034 ± 0.004 and 0.118 ± 0.008 mg/mL, respectively. EOA exhibited total antioxidant capacity of 7.299 ± 1.774 mg EAA/g. EOA exhibited potent antibacterial activity as judged by the low minimum inhibitory concentration (MIC) values against selected clinically-important pathogenic bacteria. MIC values of 6.568 ± 1.033, 5.971 ± 1.033, 7.164 ± 0.0 and 5.375 ± 0.0 μg/mL were observed against *S. aureus*, *B. subtills*, *E. coli* 97 and *E. coli* 57, respectively. EOA displayed significant antifungal activity against four strains of fungi: *F. oxysporum*, *C. albicans*, *A. flavus* and *A. niger* with values of 21.50 ± 0.43, 5.31 ± 0.10, 21.50 ± 0.46 and 5.30 ± 0.036 μg/mL, respectively. The results of the current study highlight the importance of EOA as an alternative source of natural antioxidant and antibacterial drugs to combat antibiotic-resistant microbes and free radicals implicated in the inflammatory responses accompanying microbial infection.

## 1. Introduction

Plants constitute a natural reservoir of substances with antioxidant potential [1]. The use and development of natural antioxidants are highly appreciated due to their role in the protection of human cells from damage caused by free radicals [2,3]. Natural antioxidants, rather than synthetic antioxidants, appear to be preferred by food industry users for preventing oxidative deterioration of foodstuffs caused by free radicals. It has been previously reported that the use of synthesized antioxidants such as tertbutyl hydroquinone (TBHQ), butylated hydroxytoluene, and butylated hydroxyanisole is no longer advised because of their carcinogenic potential [4]. BHA and BHT are also involved in liver damage along with other adverse health effects. Indeed, TBHQ is currently banned by some European countries and Japan [5].

Antimicrobial resistance (AMR) is a phenomenon whereby microorganisms develop a variety of strategies to combat medications designed to kill them, resulting in microbes that are resistant to treatment protocols [6]. Globally, growing attention is being allocated by scientists to AMR since it has evolved into a widespread and serious problem affecting the entire healthcare system. In addition, the World Health Organization has pointed out that AMR is the most significant concern in 2019 and has classified it among the top 10 global public health threats to humanity. The overuse of antibiotics in human medicine, animal husbandry, hygiene, and the food industry can contribute to the rise of AMR [7,8]. Fatalities attributed to AMR are alarmingly increasing and it is being projected to claim 10 million annually by the year 2050. Significant global economic losses are also expected to reach a cumulative $100 trillion if more efficient and novel therapeutic alternatives are not developed soon to contain the rapidly-evolving causative microbial agents [9]. The list of microbes that are becoming resistant to all known antibiotics is expanding, under the currently limited and insufficient repertoire of new treatments, necessitating the development of novel classes of drugs to avoid serious public health problems.

The microorganisms examined in this study are classified among the drug-resistant pathogens namely *Escherichia coli* and *Staphylococcus aureus.* As previously documented, these species are multidrug-resistant [10,11]. In addition, *Candida albicans*, which was also evaluated in this study, is known to be a drug-resistant pathogen. *Candida albicans* resistance is being widely recognized as one of the greatest expanding health burdens, owing to the widespread use of various drugs, particularly oral azoles to control this strain [12]. However, none of the currently available traditional antimycotic medications fit all of the criteria in terms of patient toxicity, ease of administration and minimal risk of resistance development.

Recently, alternative therapeutic solutions based on the exploitation of natural resources have been thoroughly researched [13,14]. In this context, the chemical constituents of the genus *Artemisia* have been the subject of numerous previous reports, which showed that this genus possesses several potentially-bioactive classes of compounds including flavonoids, polyphenols, tannins, sesquiterpene lactones and essential oil (EO) [14]. EOs from the genus *Artemisia* were reported to possess multiple biological and pharmacological effects including antimicrobial [15,16]. EOs are complex combinations of chemical molecules from various chemical families, such as aldehydes, alcohols, esters, phenols, ethers and ketones terpenes, among others. Terpenes, terpenoids, and other aromatic and aliphatic components with low molecular weights make up the majority of EOs [10,11].

The current study investigated the chemical composition of EOA along with its antioxidant and antibacterial potential against drug-resistant pathogenic microorganisms.

## 2. Materials and Methods

### 2.1. Plant Material Selection and Identification

In April 2021, *A. aragonensis* was collected from the southern slopes of Jbel Bou-Naceur in Morocco (latitude 33.59885133, longitude −3.74447934, and altitude: 1350 m). Botanical identification was conducted by a botanist under reference AHA001T7621. Thereafter, leaves were dried at room temperature for 11 days before being extracted by use of Clevenger equipment to obtain EO.

### 2.2. Extraction of Essential Oil

In the current study, hydrodistillation was used to extract EOA. Briefly, 100 g of the dried aerial parts of *A. aragonensis* were soaked in 600 mL of distilled water and boiled for 2 h using a Clevenger-type apparatus. The obtained essential oil was kept at 4 °C and its yield (%) was calculated on the basis of the dry weight of the plant material.

### 2.3. Essential Oil Chemical Identification

The EOA was characterized by GC-ULTRA apparatus equipped with VB-5 column (length: 30.00 m, internal diameter: 0.250 mm, film thickness: 0.250 μm). Operational conditions were set as follows: carrier gas (helium), injection temperature (220 °C), injection volume (1 μL), flow rate (1.4 mL/min), temperature-programmed gas chromatography (40 to 180 °C at 4 °C/min, followed by 20 min at 300 °C). The temperature of the interface was 300 °C with the following conditions: type of ionization EI (70 eV) and temperature of the ionization source (200 °C). The identification of the phytochemical components of EOA was carried out by determining their retention indices relative to a homologous series of n-alkanes and by comparing their registered mass spectra with those reported in referenced databases (NIST MS Library v.2.00) (NIST MS Library v.2.00) [17].

### 2.4. Antioxidant Activity

#### 2.4.1. Radical Scavenging Activity Test

DPPH assay was carried out according to Chebbac’s protocols [18]. To achieve this, 100 µL of EOA, at different concentrations, prepared with methanol (1.0, 0.25, 0.125, 0.0625, 0.0312, 0.0156, 0.0078, 0.0064 and 0.0019 mg/mL), were used for the testing purposes. The anti-free radical effect was measured by mixing 100 µL of each concentration previously prepared (EOA, Quercetin, Ascorbic acid and BHT) with 750 µL of DPPH (0.004%), while methanol was included as a negative control. Next, incubation was conducted in the dark for 30 min at room temperature prior to recording absorbance values at 517 nm by use of a spectrophotometer and the DPPH trapping capacity was represented as percent inhibition (Equation (1)):

PI (%) = (A0 − A/A0) × 100(1)

where PI represents the proportion of inhibition, A0 represents the negative control (methanol), and A represents the combined absorbance of DPPH and samples. All analyses were performed three times, and the findings were presented as means with standard deviations. The IC_50_ was calculated graphically by use of linear regression.

#### 2.4.2. Total Antioxidant Capacity Test (TAC)

One milliliter of a solution containing sulfuric acid, sodium phosphate, and ammonium molybdate was combined with 25 µL of each EOA concentration. The solution was then incubated for 91 min at 96 °C. The absorbance was then recorded at 695 nm against the blank with 25 µL of methanol [19]. TAC per gram of EO was expressed in milligrams of ascorbic acid equivalent (mg EAA/g). The experiment was conducted in triplicates and the obtained results were represented as means with standard deviations.

#### 2.4.3. Reducing Power Test (FRAP)

This test was carried out using the method proposed by Bourhia et al. [20]. In methanol, 500 μL of phosphate buffer solution and potassium ferricyanide were combined with 100 μL of varied doses of EOA (0.1, 0.2, 0.4, 0.8, 1.6 mg/mL). Following a 21 min incubation period, 500 μL of a 10% aqueous TCA solution, 500 μL of distilled water and 100 μL of 0.1% FeCl_3_ were added to the reaction medium. The absorbance was subsequently measured against a reagent blank containing no sample. The results were expressed as a 50% effective concentration (EC_50_).

### 2.5. Antimicrobial Activity

Antifungal activity of EOA was tested against four fungal species, including *Candida albicans* ATCC 10231, *Aspergillus niger*, *Aspergillus flavus*, and *Fusarium oxysporum*, as well as four bacterial strains, including *Escherichia coli* (ATB: 57/B6N), *Escherichia coli* (ATB: 97/BGM), *Staphylococcus aureus*, and *Bacillus subtills*, which were kindly provided by Hassan II University Hospital Center of Fez, Morocco.

#### 2.5.1. Disk Diffusion Method

In the present study, the disk diffusion method was used to evaluate antifungal and antibacterial activity of EOA [21]. For this purpose, bacteria were grown in Petri plates having nutrient broth medium (NB), whereas fungal strains were grown in Petri dishes possessing a malt extract agar (MEA) medium. From fresh bacteria culture, a few colonies were aseptically seeded in 0.9% sodium chloride (NaCl) at a density of 0.5 McFarland (10^7^ to 10^8^ CFU/mL), whilst the yeast suspension was determined to be approximately 10^6^ CFU/mL. After being soaked in 10 µL of EOA, 6 mm diameter disks were placed on the surface of petri dishes. [15,22,23]. Next, the inoculated Petri dishes were incubated in the dark at 30 °C and 37 °C for the fungal and bacterial species, respectively. The inhibition rate, expressed in percentages, was calculated 24 and 48 h post-incubation for bacteria and *C. albicans*, respectively, and 7 days post-inoculation for *A. niger*, *A. flavus* and *F. oxysporum* [18]. The growth inhibition zones were determined in mm.

#### 2.5.2. Determination of the Minimum Inhibitory Concentration (MIC)

The microdilution method, which was originally published in Balouir’s earlier work [24], was undertaken to determine the MIC of EOA against bacterial and fungal strains. In this context, the MIC was calculated by direct observation of growth in the wells using the colorimetric method (TTC 0.20 percent (*w*/*v*)) after an incubation period of 24 h for bacteria at 37 °C, 48 h for yeast and seven days for fungi at 30 °C.

### 2.6. Statistical Analysis

The obtained findings in this research work were expressed as means with standard deviations of triplicate tests. Normality was checked by use of the Shapiro–Wilks test and the assumption of homogeneity of variance was evaluated using Levene’s test. The non-parametric Tukey’s statistical test was employed as a post-hoc test for multiple comparisons. When *p* < 0.05, a statistically significant difference was considered.

## 3. Results and Discussion

### 3.1. Essential Oil Yield

The yield of EOA was 1.18%, which was reasonable compared with EOs extracted from plants that have been industrially exploited as a natural source of EOs. In this context, species among genus *Artemisia* were used for comparison purposes including Artemisia *frigida* (1.5%) and *Artemisia cana* (1.3%). By contrast, species of *A. absinthium*, *A. dracunculus, A. biennis, A. ludoviciana* and *A. longifolia* were found to produce lower EO yield than *A. aragonensis* [25]. The observed difference can be explained by the environmental and edaphic factors, extraction technique, drying, harvesting period and cultural practices that influence both quality and quantity of compounds in plants [26].

### 3.2. Chemical Composition Identification of the Essential Oil

**Figure 1:**
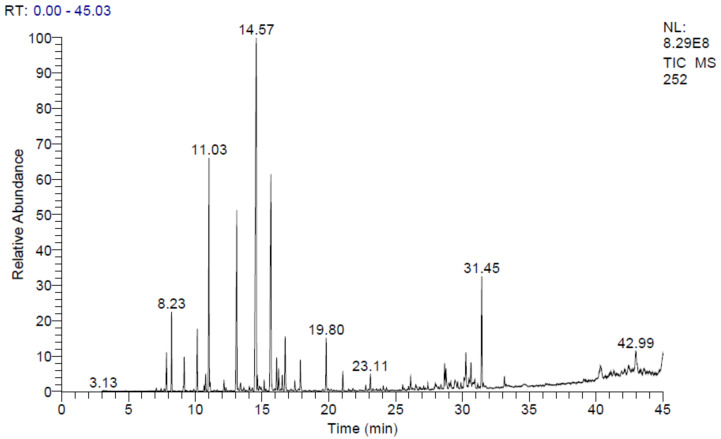
Chromatographic profile of EOA profiled by GC-MS.

**Figure 2:**
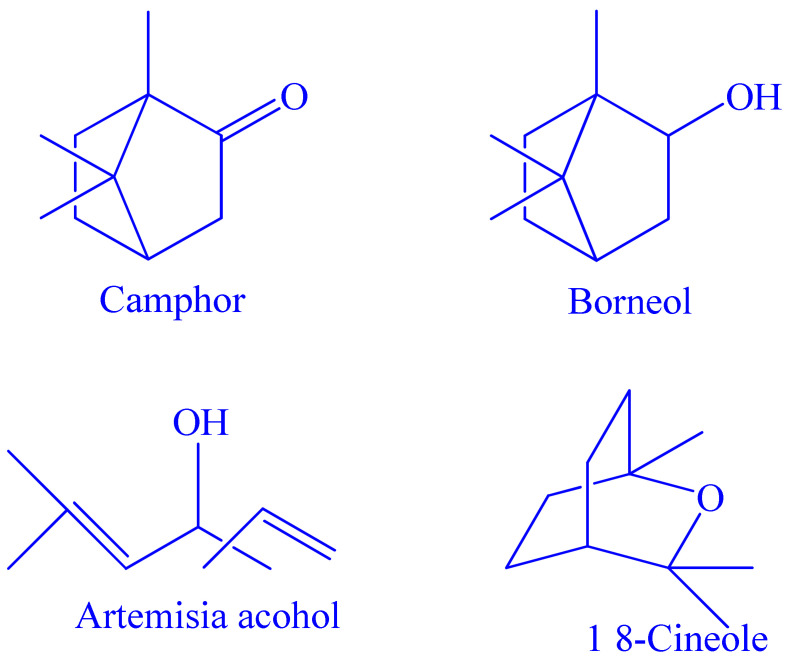
Molecular structure of some major phytochemicals identified in EOA.

**Figure 3:**
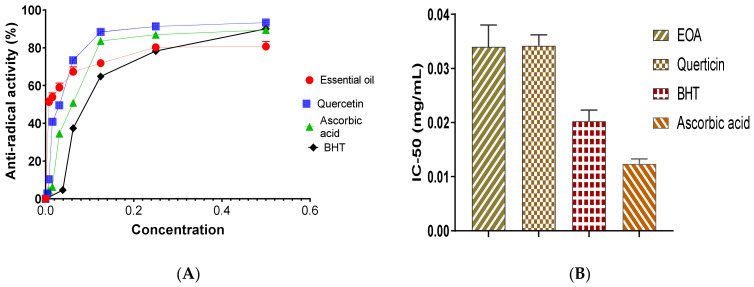
(**A**) Anti-radical activity of EOA and controls (BHT, Ascorbic Acid and Qercetin) by use of DPPH assay. (**B**) IC_50_ values of anti-radical activity of EOA and controls (BHT, Ascorbic Acid and Qercetin).

The chromatographic analysis of EOA showed the presence of 34 compounds representing 99.96% of the EO (Figure 1). In this context, GC-MS results demonstrated that EOA consisted of Camphor (24.97%), Borneol (13.20%), 1.8 Cineol (10.88%), Artemisia alcohol (10.20), α-Bisabolone oxide A (5.63%) and Camphene (3.10%) (Table 1, Figure 2 and Figure 3).

The detected components were classified according to functional categories and the results revealed that oxygenated monoterpenes (70.14%) were the most abundant in EOA. These findings matched those reported in previous studies [16], which reported the richness of EOA native to Spain, in Camphor (15%), Cineol (13.3%), Borneol (4.8%) and Camphene (1.9%). The chemical composition, particularly amounts of monoterpene alcohols, found in EOA in our study is similar to those reported in Israeli species. However, the chemical composition of EOA was different when compared to Algeria and Tunisia cultivars in terms of chemical content, notably monoterpene alcohols [23,24]. For a more detailed comparison, the chemical content of EOs extracted from the aerial parts of four Artemisia species, *A. cana*, *A. frigida*, *A. longifolia and A. ludoviciana*, growing in *Canada* was higher in 1,8-cineole (21.5–27.6%), davanone (11.50%) and camphor (15.9–37.3%). EO of *A. absinthium* was found to be rich in myrcene (10.80%), trans-sabinyl acetate (26.40%) and trans-thujone (10.1%). *A. biennis* contained more (E)-β-farnesene (40%), (Z)-β-ocimene (34.7%), acetylenes (11.00%) (Z)- and (E)-en-yn-dicycloethers. Phenylpropanoids (16.2%) and methyl eugenol were the primary components of the EO from *A. dracunculus* (35.8%) [27,28].

Several studies indicated that *A. aragonensis* was characteristically distinguished by the presence of potentially bioactive compounds including chrysantenone and davanone [26,29,30], which are absent in our plant that was collected from the southern slope of Jbel Bou-Naceur. Therefore, this difference in chemical composition can be an indicator of the difference in the ecosystem diversity where species grow. Moreover, our results showed that EOA contained artemisia alcohol and artemisia acetate, which can be used as a distinctive indicator of *A. aragonensis* from the southern slope of Jbel Bou-Naceur of the folded Middle Atlas of Morocco.

### 3.3. Antioxidant Activities

#### 3.3.1. Test DPPH

The ability of EOA to scavenge the DPPH free radical was used to assess its anti-radical activity (Figure 3A). Figure 3B shows the obtained results of tests measuring the percentage of DPPH inhibition as well as the IC50 values. In this respect, the results indicated that the EOA was capable of inhibiting the DPPH free radicals with an IC_50_ value of 0.034 ± 0.004 mg/mL, whilst other tested synthetic antioxidants such as BHT, Ascorbic Acid, and Quercetin showed IC_50_ values of 0.0203 ± 0.005, 0.0124 ± 0.001 and 0.0342 ± 0.002 mg/mL, respectively. Furthermore, when compared to other EOs from Artemisia species tested by the same bioassays such as *A. absinthium*, *A. biennis*, *A. cana*, *A. longifolia*, *A. dracunculus*, *A. frigida* and *A. ludoviciana*, the EOA exhibited the strongest radical-scavenging activity [31]. Therefore, it can be concluded that EOA under investigation in the current study possesses a significant antioxidant capacity that is superior to EOs extracted from the other species in the genus *Artemisia*.

The EOA was higher in oxygenated monoterpenes (Table 1), which can act as radical scavengers. Our results are in agreement with other reports that documented that the high antioxidant effects of EOs are attributed to their richness in oxygenated monoterpenes and/or sesquiterpenes [32]. Terpenes are a type of natural chemical derived from plants; they are generated by the condensation of isoprene units (C5H8) and are divided into monoterpenes (C10) and sesquiterpenes (C15). Terpenoids are oxygen-containing compounds of these plants [33]. Moreover, several studies have documented that EOs possessing minimal phenolic components may exhibit antioxidant properties [34]. Furthermore, camphor, which is a major constituent in our essential oil (24.97%), has been reported to have a potent antioxidant activity [35]. Typically, the chemical characterization of several EOs has indicated the existence of only 2–3 primary components at relatively high concentrations (20–70%) relative to other constituents contained in minute quantities [36].

The lipid peroxidation process, which is characterized by a radical chain reaction, produces peroxyl radicals as intermediates. Antioxidants, including terpenes break the chain and react with the lipid peroxyl radicals, and thereby interrupt this process. By reacting with another radical, the antioxidant radical can be eliminated, resulting in a stable product [37]. The presence of antioxidants in terpenoids causes the reduction of the Fe3+ or ferricyanide complex to its ferrous form [38]. Polyphenols with hydroxyl groups possessed powerful antioxidant activity, which was due to their ability to release more atoms to stabilize free radicals [39]. Generally, antiradical activity depends on the number, position and nature of substituents on the B and C rings (hydroxylated, methoxylated, glycosylated groups) and the degree of polymerization [40].

#### 3.3.2. Total Antioxidant Capacity

In the present work, the total antioxidant capacity (TAC) content of EOA was obtained by use of an ascorbic acid calibration curve. The obtained results revealed that the examined EOA had considerable antioxidant potency equivalent to 7.299 ± 1.774 EAA/g EOA. Earlier studies identified a strong link between the chemical content of EOs and their antioxidant activity, particularly when molecules possess hydroxyl functionalities [41]. In this context, it was reported that EOs that are rich in oxygenated monoterpenes and phenolic compounds possess high antioxidant potency, which conforms to our present findings revealing the richness of EOA in oxygenated monoterpenes [42].

TAC exhibited by EOA is likely to be attributed to some chemical constituents that were identified by GC-MS (Figure 1 and Figure 2), particularly pulegone [43]. It is well documented in the literature that the antioxidant effect of EOs prepared from aromatic plants are mostly due to active molecules, particularly monoterpenes, ketones menthone, and isomenthone [44,45,46]. As a result, EOs with a higher terpene content have been reported to possess powerful antioxidant potential [35,36]. Minor compounds in EOs are more likely than major chemicals to play a significant role in the observed antioxidant [47].

#### 3.3.3. Ferric Reducing Antioxidant Power Assay

**Figure 4:**
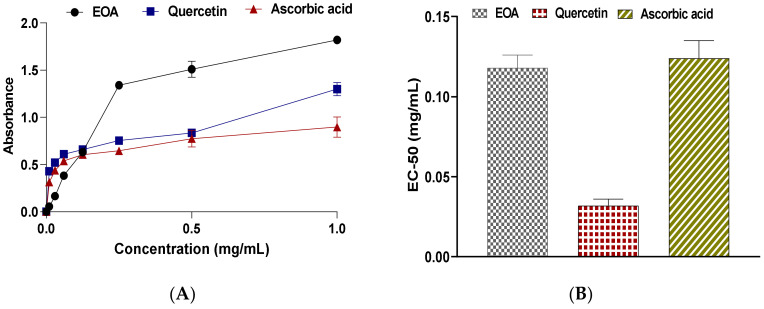
(**A**) Ferric reducing antioxidant power of EOA and controls (BHT, Ascorbic Acid and Qercetin). (**B**) IC_50_ values of Ferric reducing antioxidant power of EOA and controls (BHT, Ascorbic Acid and Qercetin).

The findings of the FRAP test (Figure 4A,B) revealed that the EOA resulted in a dose-dependent reduction in antioxidant power with an EC_50_ value of 0.118 ± 0.008 mg/mL. EOA had a powerful antioxidant effect when compared to the EC_50_ obtained with the positive control quercetin (EC_50_ = 0.032 ± 0.004 mg/mL) and ascorbic acid (EC_50_ = 0.124 ± 0.011 mg/mL). The reducing power of the EOA extracted from the studied plant was probably due to the presence of chemically bioactive compounds such as Camphor, Borneol, 1.8 Cineol, Artemisia alcohol, α-Bisabolone oxide and camphene, which can serve as electron donors to scavenge free radicals [48]. Therefore, it can be confirmed that the EOA under investigation has a very significant antioxidant capacity.

It has been suggested that the antioxidant effect of EOs is mostly attributed to their chemical constituents possessing hydroxyl functionalities [49]. As a result, EOs higher in terpene content have more antioxidant power, which is in agreement with our GC-MS results, indicating the richness of EOA in terpenes. Moreover, results of TAC were in accordance with the literature [50], where it was reported that EOs from species among Genus Artemisia possessed antioxidant power, particularly *Artemisia annua* L., *Artemisia judaica* L. and *Artemisia vulgaris* L.

### 3.4. Antibacterial and Antifungal Activity of EOA

#### 3.4.1. Antibacterial Activity of Essential EOA

**Figure 5:**
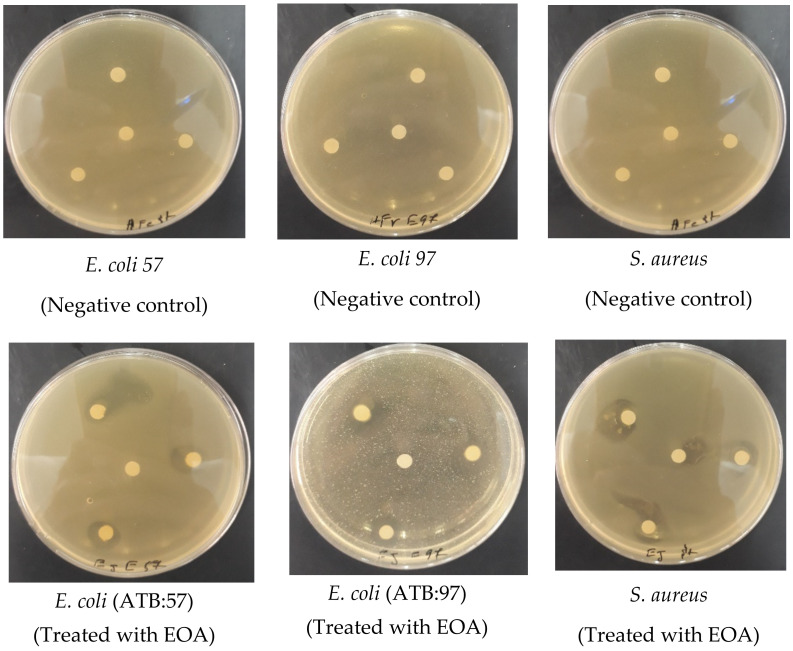
Photographs displaying the effects of EOA on the tested bacteria.

The results of the antibacterial effect of EOA, including the inhibition diameter and minimum inhibitory concentration (MIC) are summarized in Table 2 and Table 3 and Figure 5. EOA showed significant antibacterial effects against all bacterial strains tested, whether Gram-negative (*E. coli* ATB: 57, *E. coli* ATB: 97) or Gram-positive (*B. Subtilis* and *S. aureus*) (*p* > 0.05). Although these bacteria are known to be highly virulent and pathogenic, they were found to be sensitive to EOA.

The observed antibacterial effect exhibited by EOA was significant compared to the positive antibiotic controls Streptomycin and Ampicillin that have been shown to be generally ineffective against most of the tested strains, with neither inhibition zones nor bactericidal effect being observed (*p* < 0.05). The results presented here clearly document the development of antibacterial resistance by bacterial strains, which is in agreement with previous studies [17,51]. Additionally, our findings were consistent with those reported elsewhere [50], which showed that the EO extracted from *Withania frutescens* L. possessed significant antibacterial effects against *S. aureus*, *E. coli* 57 and *E. coli* 97.

Our findings were consistent with previous results [51,52], which showed that the EOA from Tunisia had high efficacy against *E. coli* (11.30 mm) and *B. cereus* (23 mm), as well as *P. aeruginosa* PAA1. Moreover, previous work showed significant activity of the genus *Artemisia* against *S. aureus* SASMA1 (17.70 mm). The antibacterial activity of EOA could be due to Borneol, 1,8 cineol and Artemisia alcohol identified by GC-MS [52]. Our results were in agreement with a previous study that attributed the antibacterial power of EOA to the presence of a significant amount of Camphor [52], which can confirm that the oxygenated monoterpenes possess antibacterial power against several bacteria [53,54]. The mechanism of action (MOA) of Bornoel, Artemisia alcohol and 1,8-cineole is most likely due to their ability to form hydrogen bonds, which determines their activity towards Gram positive bacteria [55]. Essential oils rich in terpenes including camphor can penetrate cell walls and the cytoplasmic membrane, inducing polysaccharide structure, fatty acid, and phospholipid permeability disorders [56]. Since camphor is the most predominant component in EOA (24.97%; Table 1), it could be responsible for the observed antimicrobial effect of EOA. The molecular interaction of the functional groups of the components of EOA with the bacteria’s wall, which creates deep lesions, could explain EOA’s antibacterial effectiveness. It’s also possible that this activity is the consequence of a synergistic effect of various components of EOA [57].

The antibacterial capabilities of EOA documented in the current study can be explained by the lipophilic nature of the oil, which allows it to easily infiltrate bacterial cells and kill them. In this sense, it has been claimed that hydrocarbons make EOs preferentially lodge in biological membranes leading to the disruption of membrane permeability and eventually triggering rapid death of microorganisms [46,47]. Phytochemicals (Camphor, Borneol, 1,8-cineole, Artemisia alcohol, -Bisabolone oxide, and Camphene) in the oil can function in synergy more than individually since previous studies have demonstrated that the antibacterial activity of EOs was shown to be greater than its individually examined constituents [58,59,60]. In order to have an antibacterial effect, antimicrobial agents must reach and interact with target microorganism-specific sites. In bacteria, the drug–target interaction is commonly disrupted by a variety of resistance mechanisms, resulting in ineffectiveness of antimicrobial drugs and ultimately aiding the development of bacterial strains that are resistant to the examined agents [61,62]. However, EOs can easily permeate cell walls and cytoplasmic membranes due to their lipophilic nature, which leads to bacterial death by disrupting polysaccharide structure, fatty acids and phospholipids [63]. EOA has essentially the same efficacy against Gram-positive and Gram-negative bacteria, according to our results, and therefore, it has great potential as a powerful broad-spectrum weapon to control pathogenic and multidrug-resistant strains.

#### 3.4.2. Antifungal Activity of the Essential Oil

**Figure 6:**
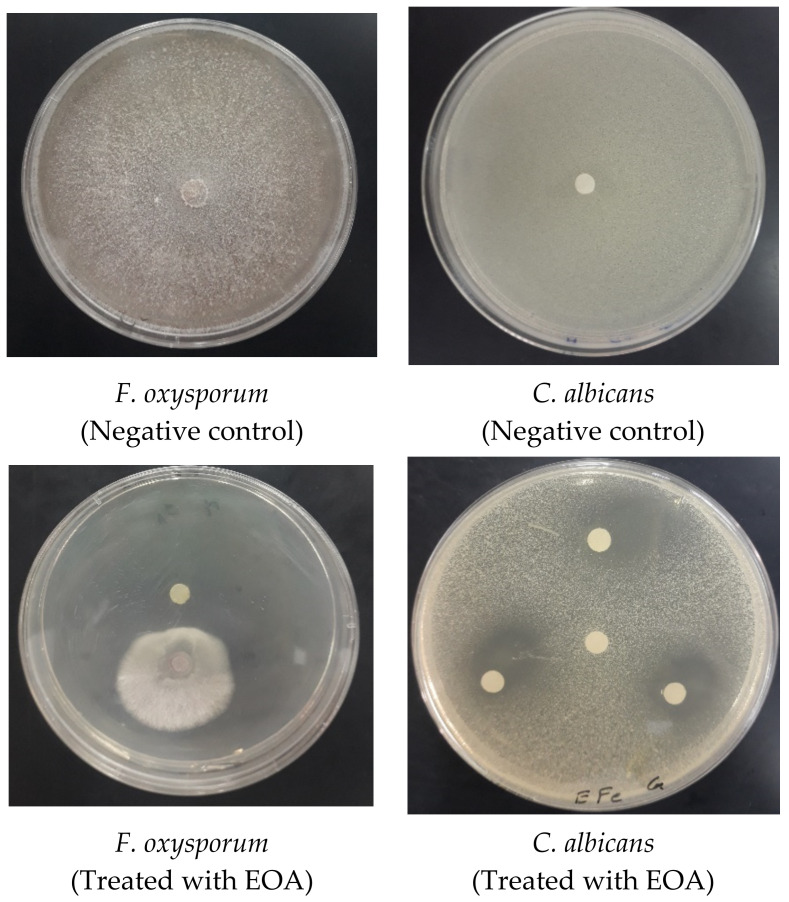
Photographs displaying the effects of EOA on the tested fungi.

In vitro testing of EO derived from *A. aragonensis* against *A. niger*, *A. flavus*, *F. oxsporum* and *C. albicans* revealed promising antifungal activity with inhibition zones of 68.51 ± 1.06; 71.72 ± 0.52, 46.50 ± 1.01 and 40.00 ± 1.00 mm, respectively. Additionally, the MIC values observed for EOA against *A. niger*, *A. flavus* and *F. oxysporum* were 21.50 ± 0.43, 5.31 ± 0.10 and 21.50 ± 0.46 µg/mL, respectively (Table 4 and Figure 6). EOA was more effective towards *A. flavus* (MIC = 21.50 ± 0.46 μg/mL) and *C. albicans* (MIC = 5.31 ± 0.10 μg/mL) when compared to *A. niger* (MIC = 5.30 ± 0.036 μg/mL) and *F. oxysporum* (MIC = 21.50 ± 0.43 μg/mL) (*p* < 0.05). Regardless of the dose used for testing, EOA significantly inhibited fungal growth compared to the control antifungal pharmaceutical drug Fluconazole (*p* < 0.05). These findings agreed with those reported in earlier works [51], which showed that the EO from *Withania frutescens* L. possess antifungal effects against *C. albicans*, with a MIC value of 4 µg/mL.

Several studies have been devoted to the control of *A. niger*, *A. flavus*, *Fusarium oxysporum* and *C. albicans* by the use of natural products including the study by El Barnossi et al. [10], which demonstrated that the *Bacillus* sp Gn-A11-18 had antifungal activity against *C. albicans* and *A. niger.* Bulgasem’s study also reported that cell-free supernatant produced by *Lactobacillus plantarum* isolated from vegetables has strong antifungal activity against *C. albicans* [31].

Results of the present work indicated significant antifungal activity of EOA against *Aspergillus flavus*, which is classified as a saprophyte in soils worldwide. This fungal strain has been reported to inflect a serious burden loss on cash crops including peanuts, corn and cottonseed during both pre-and post-harvest conditions [45,46,47]. *A. flavus* fungus has also been associated with human and animal diseases, either through invasive growth causing aspergillosis or through consumption of contaminated food causing aflatoxicosis, which is often fatal in immunocompromised humans [48].

The antimicrobial MOA of EOs is multifaceted, and it is determined by their chemical makeup and quantities of the prominent single compounds present. Numerous studies have revealed insightful data on the MOA of the observed antifungal activity exhibited by EOs. The MOAs of the observed EO-mediated antifungal and antibacterial effects are analogous to one another. A large number of studies have found that the phytochemicals present in EOs disrupt cell membranes and alter a variety of other cellular functions, including production of energy [63]. Reduced membrane potentials, interruption of proton pumps and ATP exhaustion may all contribute to the observed antimicrobial activity [64]. The coagulation of cell content, potassium ion efflux, cytoplasm leakage, and finally cell apoptosis or necrosis, which leads to cell death, are all biochemical hallmarks of the noted antimicrobial activity of EOs. In this context, it was reported that EOs rich in thymol and p-cymene easily permeate fungal cells causing membrane damage [65]. The fungicidal effect of natural agents is attributed to direct damage to the cell membrane rather than metabolic impairment, eventually leading to the execution of fungal death [66]. This cytotoxic effect can be linked to monoterpenes present in EOs, as it might potentially operate as a cell membrane solvent. Similarly, previous literature revealed that the fungicidal activity of an EO rich in thymol and p-cymene against *Candida* spp. was due to direct cytoplasmic membrane disruption [31].

## 4. Conclusions

The outcome of this work clearly indicated that the essential oil extracted from *A. aragonensis* had excellent antibacterial and antifungal potencies against clinically important drug-resistant pathogenic microorganisms. These finding are intriguing since they suggest that EOA could potentially be employed as an alternative to traditional antimicrobial treatment. Camphor, borneol, 1,8-cineole, artemisia alcohol, α-bisabolone oxide and camphene were identified in the EO of the investigated plant, which could be responsible for the recorded activity. Although the MOA of EOA is still being investigated, it is well recognized that a complex mixture of constituents can potentially have multiple biological responses concurrently. Hence, future investigation will concentrate on determining the MOA of single purified chemicals. Prior to any prospective application of EOA as a natural medication to control microorganisms, assessment of the potential undesirable impacts on non-target organisms along with pre-clinical and clinical studies on non-human primates and humans will be essential.

## Figures and Tables

**Table 1:** Phytochemical components identified in EOA by GC-MS.

	RI					
Area (%)	Lit	Obs	Chemical Classes	Compound Name	R.T (min)	P
1.53	933	933	MO.H	α-Pinene	7.84	1
3.1	949	948	MO.H	Camphene	8.23	2
1.43	980	982	MO.H	β-Pinene	9.17	3
2.75	999	998	MO.O	Yamogi alcohol	10.15	4
0.69	1026	1024	MO.H	o-Cymene	10.78	5
10.88	1032	1030	MO.O	1,8-Cineole	11.03	6
0.48	1017	1019	MO.H	α-Terpinene	12.15	7
10.2	1083	1089	MO.O	Artemisia alcohol	13.11	8
0.51	1102	1107	MO.O	Thujone	13.39	9
24.97	1146	1151	MO.O	Camphor	14.57	10
0.52	1139	1145	MO.O	Trans-pinocarveol	14.65	11
0.44	1164	1163	MO.O	Pinocarvone	15.16	12
13.2	1169	1171	MO.O	Borneol	15.66	13
1.39	1082	1084	MO.O	Terpinen-4-ol	16.09	14
1	1173	1178	O	Artemisia acetate	16.24	15
0.69	1133	1137	MO.O	α–Terpineol	16.51	16
2.73	1198	1195	MO.O	Myrtenol	16.73	17
0.42	1216	1220	MO.O	Trans-Carveol	17.46	18
1.44	1237	1239	MO.O	Pulegone	17.87	19
2.33	1288	1286	O	Bornyl acetate	19.8	20
0.83	1326	1327	O	Myrtenyl acetate	21.05	21
0.75	1376	1372	SQ.H	α-Copaene	23.11	22
0.71	1485	1480	SQ.H	Germacrene D	26.12	23
1.26	1578	1579	SQ.O	Spathulenol	28.66	24
1.26	1586	1583	SQ.O	Caryophyllene oxide	28.77	25
0.5	1624	1625	SQ.O	Isospathulenol	30.12	26
2.2	1632	1633	SQ.O	γ-Eudesmo	30.26	27
0.51	1640	1642	SQ.O	Cadinol	30.5	28
1.3	1650	1652	SQ.O	β-Eudesmo	30.64	29
0.45	1658	1657	SQ.O	Bisabolol oxyde B	30.93	30
5.63	1685	1688	SQ.O	Bisabolone oxide A	31.45	31
0.56	1749	1751	SQ.O	α-Bisabolol oxide A	33.14	32
1.33	2800	2804	O	Octacosane	40.32	33
1.63	2500	2503	ST.H	Pentacosane	42.99	34
	Chemical classes					
7.23	Monoterpene hydrocarbons (MO.H)					
70.14	Oxygenated monoterpenes (MO.O)					
1.46	Sesquiterpene hydrocarbons (SQ.H)					
13.67	Oxygenated sesquiterpenes (SQ.O)					
1.63	Sesterpene (ST.H)					
5.49	Other compounds (O)					
99.62	Total identification					

P: Peak; R.T: Retention time; Obs: Observed; Lit: Literature; R.I: Retention index; MO.H: Monoterpene hydrocarbons; MO.O: Oxygenated monoterpenes; SQ.H: Sesquiterpene hydrocarbons; SQ.O: Oxygenated sesquiterpenes; ST.H: Sesterpene; O: Other compounds.

**Table 2:** Inhibition zones induced by EOA and controls (Streptomycin and Ampicillin) vs. bacterial strains (mm).

Compound	Gram-Negative Bacteria		Gram-Positive Bacteria	
	*E. coli* (ATB:57)	*E. coli* (ATB:97)	*S. aureus*	*B. subtilis*
Essential oil	13.00 ± 0.00 ^a^	13.67 ± 1.15 ^a^	14.67 ± 0.58 ^a^	13.33 ± 0.58 ^a^
Streptomycin	_	_	9.11 ± 0.43	_
Ampicillin	_	_	_	_

Row values with the same letter (a) did not differ significantly (means ± SD, *n* = 3, one-way ANOVA; Tukey’s test, *p* ≤ 0.05).

**Table 3:** Minimum inhibitory concentration induced by EOA and controls (Streptomycin and Ampicillin) vs. bacterial strains (µg/mL).

Compound	Gram-Negative Bacteria		Gram-Positive Bacteria	
	*E. coli* (ATB:57)	*E. coli* (ATB:97)	*S. aureus*	*B. subtilis*
EOA	5.375 ± 0.00 ^a^	5.971 ± 1.033 ^a^	6.568 ± 1.033 ^a^	7.164 ± 0.0 ^a^
Streptomycin	0.25 ± 0.00 ^a^	0.5 ± 0.00 ^b^	0.062 ± 0.00 ^c^	_
Ampicillin	_	_	_	_

Row values with the same letters (a, b or c) did not differ significantly (means ± SD, *n* = 3, one-way ANOVA; Tukey’s test, *p* ≤ 0.05).

**Table 4:** Evaluation of the antifungal activity of EOA and Fluconazole by use of inhibition zone and minimum inhibitory concentration (MIC).

	Inhibition Diameter (mm)		Minimum Inhibitory Concentration (µg/mL)	
Fungal Strains	EOA	Fluconazole	EOA	Fluconazole
*A. niger*	68.51 ± 1.06 ^a^	36.12 ± 3.70 ^b^	21.50 ± 0.43 ^c^	2.01 ± 0.01 ^d^
*A. flavus*	71.72 ± 0.52 ^a^	29.41 ± 5.07 ^b^	5.31 ± 0.10 ^c^	1.21 ± 0.01 ^d^
*F. oxysporum*	46.50 ± 1.01 ^a^	39.52 ± 2.16 ^a^	21.50 ± 0.46 ^a^	1.82 ± 0.01 ^d^
*C. albicans*	40.00 ± 1.0 ^a^	33.08 ± 4.17 ^a^	5.30 ± 0.036 ^c^	3.12 ± 0.20 ^d^

Row values with the same letters (a, b, c or d) did not differ significantly (means ± SD, *n* = 3, one-way ANOVA; Tukey’s test, *p* ≤ 0.05).

## Data Availability

All data reported here is available from the authors upon request.

## Sample Availability

Samples of the compounds are available from the authors.
